# Predicting monthly hospital outpatient visits based on meteorological environmental factors using the ARIMA model

**DOI:** 10.1038/s41598-023-29897-y

**Published:** 2023-02-15

**Authors:** Lu Bai, Ke Lu, Yongfei Dong, Xichao Wang, Yaqin Gong, Yunyu Xia, Xiaochun Wang, Lin Chen, Shanjun Yan, Zaixiang Tang, Chong Li

**Affiliations:** 1 Department of Biostatistics, School of Public Health, Medical College of Soochow University, Suzhou, 215123 China; 2 Jiangsu Key Laboratory of Preventive and Translational Medicine for Geriatric Diseases, Medical College of Soochow University, Suzhou, 215123 China; 3 Department of Orthopedics, Affiliated Kunshan Hospital of Jiangsu University, No. 91 West of Qianjin Road, Suzhou, 215300 Jiangsu China; 4 Information Department, Affiliated Kunshan Hospital of Jiangsu University, Suzhou, 215300 Jiangsu China; 5 Meteorological Bureau of Kunshan City, Suzhou, 215337 Jiangsu China; 6 Ecology and Environment Bureau of Kunshan City, Suzhou, 215330 Jiangsu China

**Keywords:** Environmental impact, Risk factors, Statistics

## Abstract

Accurate forecasting of hospital outpatient visits is beneficial to the rational planning and allocation of medical resources to meet medical needs. Several studies have suggested that outpatient visits are related to meteorological environmental factors. We aimed to use the autoregressive integrated moving average (ARIMA) model to analyze the relationship between meteorological environmental factors and outpatient visits. Also, outpatient visits can be forecast for the future period. Monthly outpatient visits and meteorological environmental factors were collected from January 2015 to July 2021. An ARIMAX model was constructed by incorporating meteorological environmental factors as covariates to the ARIMA model, by evaluating the stationary ${R}^{2}$, coefficient of determination ${R}^{2}$, mean absolute percentage error (MAPE), and normalized Bayesian information criterion (BIC). The ARIMA ${(0, 1, 1) (0, 1, 0)}_{12}$ model with the covariates of $\text{SO}_{2}$, ${PM}_{2.5}$, and $\text{CO}$ was the optimal model. Monthly outpatient visits in 2019 can be predicted using average data from past years. The relative error between the predicted and actual values for 2019 was 2.77%. Our study suggests that $\text{SO}_{2}$, ${PM}_{2.5}$, and $\text{CO}$ concentration have a significant impact on outpatient visits. The model built has excellent predictive performance and can provide some references for the scientific management of hospitals to allocate staff and resources.

## Introduction

Due to the rapidly aging population growth, China’s healthcare resources are relatively scarce and unevenly distributed, creating a huge gap between supply and demand^1^. There is a growing interest in the optimal management of healthcare resources, which makes accurate forecasting of future healthcare demand and resource availability even more critical. The pressure on outpatient clinics is increasing every year due to the increase in the number of patients and the growing complexity of health conditions. The ability to forecast outpatient visits is critical to avoid overcrowding and provide quality patient care. Accurate and reliable forecasting of the number of different types of outpatient visits helps to scientifically allocate key medical resources such as medical equipment, and hospital beds^1^. So, accurate forecasting of outpatient visits is beneficial for the reasonable planning and allocation of healthcare resources to meet medical needs or anticipate potential medical resource shortages.

Some meteorological environmental factors influence the number of outpatient visits^2,3^. The distributed lag nonlinear model (DLNM) and generalized linear model (GLM) models have been commonly used in previous studies to explore the relationship between meteorological environmental factors and outpatient visits. The DLNM is a model that can accommodate both nonlinear exposure–response relationships and the lagged effects of exposure factors. It is more suitable for studying the effects of meteorological factors, air pollution, or environmental conditions on human health^4^. Some previous studies using the DLNM model showed that DTR (diurnal temperature range) had a significant impact on outpatient visits for the common cold^5^. The temperature had a significantly negative effect on the number of daily outpatients. Daily outpatient visits for eczema were found to have strong positive associations with changes in ${PM}_{10}$ levels^6^. The GLM model was also applied to analyze the exposure–response relationship between air pollutants and daily outpatient visits. GLM model can combine the time-series regression analysis with the family of Poisson distribution and natural splines, and estimates both short-term and long-term relationships between air pollutants and outpatient visits. For example, Wang et al. found at lag 0 day, the RR of respiratory outpatients increased by 0.37% with a 10 ${\upmu {\text{g}}/\text{m}}^{3}$ increase in ${PM}_{2.5}$, $\text{O}_{3}$ was not significantly associated with respiratory outpatient visits during the warm periods, but was negatively associated during the cold periods^7^. However, neither the DLNM model nor the GLM model can predict the number of outpatient visits.

Some deep-learning models can be used for outpatient visit prediction. The LSTM (long short-term memory) is an attractive method and has been used for outpatient visit forecasting studies in recent years^8^. However, the main drawback of LSTM is that it has a complex training model that is prone to overfitting and requires a long training time. Also, LSTM cannot be used to select important predictors. Random forest (RF) and extreme gradient boosting (XGBoost) are used in the proposed two-dimensional hierarchical influenza outpatient visit forecasting model and could provide effective forecasting results. However, it is too complicated to construct a two-dimensional hierarchy and consider the region values for model construction^9^.

The ARIMA model is relatively simple and can also be used to predict the number of outpatient visits without using covariates^1^. Some previous studies have used covariates, but first, need to obtain covariate data for that moment and do not serve to predict the future. For instance, the ARIMA model with the covariates of atmospheric pressure, wind speed and mean temperature in 2015 was adopted to predict the incidence of brucellosis in 2015^10^. Some studies also examine the effect of independent covariate delay effects on outpatient visits^10,11^.

In this study, we retrospectively analyze the time series of outpatient visits in Kunshan from January 2015 to July 2021 using an ARIMA model. To explore the relationship between meteorological environmental factors and outpatient visits, also to develop a simple and practical model that can be used to predict outpatient visits.

## Material and methods

### Study site and data collection

Kunshan City is part of Suzhou, Jiangsu Province, China. Kunshan City is located southeast of the Yangtze River Delta (121°E, 31°N) and belongs to the northern subtropical monsoon maritime climate zone. It has a warm, humid, and rainy climate, four distinct seasons, and abundant light and rainfall. Its annual average temperature is 17.6 °C, the annual average precipitation is nearly 1200.4 mm, and the annual average sunshine time is about 1789.2 h. The population of Kunshan City grew from 787 thousand in 2015 to 1.067 million in 2020.

Meteorological data for Kunshan, including monthly average atmospheric pressure (hPa), monthly average temperature (°C), monthly average relative humidity (%), monthly average rainfall (mm), monthly 2-min average wind velocity (m/s), monthly average extreme wind speed (m/s), monthly average sunshine hours (h), were provided by the Meteorological Bureau of Kunshan City, Suzhou, Jiangsu Province. Environmental data, including $\text{SO}_{2}$ (${\upmu {\text{g}}/\text{m}}^{3}$), $\text{NO}_{2}$ (${\upmu {\text{g}}/\text{m}}^{3}$), ${PM}_{2.5}$ (${\upmu {\text{g}}/\text{m}}^{3}$), ${PM}_{10}$ (${\upmu {\text{g}}/\text{m}}^{3}$), $\text{CO}$ (${\upmu {\text{g}}/\text{m}}^{3}$), and $\text{O}_{3}$ (${\upmu {\text{g}}/\text{m}}^{3}$), were provided by the Ecology and Environment Bureau of Kunshan City, Suzhou, Jiangsu Province.

We collected outpatient data from the Affiliated Kunshan Hospital of Jiangsu University from January 2015 to July 2021 as our study subjects. The data were aggregated as secondary data without any personal information, and therefore do not require informed consent. There were no missing values in this data set.

### Statistical analysis

The ARIMA (Autoregressive Integrated Moving Average) model is the most frequently used method in time series analysis, based on the Box-Jenkins Model (1960), and can be used to predict the future values of time series using past values and can also analyze the multiple relationships between the independent and dependent variables^12,13^. ARIMA model was composed of autoregression (AR) with a lag number denoted by p, integrate (I) with a lag number denoted by d, and moving average (MA) with a lag number denoted by q. AR indicates that current observations are correlated with previous ones, which provides a possibility of predicting diseases with a time trend. MA refers to the correlation between the errors as well as the weighted average of random disturbance terms^14^. Because the monthly outpatient visits in this study exhibited seasonality, the seasonal autoregressive integrated moving average model (SARIMA or seasonal ARIMA) was used. SARIMA model includes the seasonal characteristics of time series and could account for seasonal autocorrelations and trends adequately^12,14,15^. The SARIMA model can be expressed as SARIMA ${(\mathrm{p},\mathrm{ d},\mathrm{ q}) (\mathrm{P},\mathrm{ D},\mathrm{ Q})}_{s}$ or ARIMA ${(\mathrm{p},\mathrm{ d},\mathrm{ q}) (\mathrm{P},\mathrm{ D},\mathrm{ Q})}_{s}$. The parameter P represents seasonal autoregression, D represents seasonal differencing, Q represents the seasonal moving average, and s represents the seasonal cycle. The time series of ${Y}_{t}$ could be written as follows:

1 $${Y}_{t}=\frac{{\theta }_{q}(B){\Theta }_{Q}({B}^{S}){a}_{t}}{{\upphi}_{P}({B}^{S}){\varphi }_{p}(B){(1-B)}^{d}{(1-{B}^{S})}^{D}}$$

where ${Y}_{t}$ is the predicted outpatient visits at time t, ${\varphi }_{p}\left(B\right)$ is the operator of the autoregressive model, ${\theta }_{q}\left(B\right)$ is the operator of the moving average model, ${(1-B)}^{d}$ is the component of the ordinary differences, ${\upphi}_{P}({B}^{S})$ is the operator of the seasonal autoregressive model, ${\Theta }_{Q}({B}^{S})$ is the operator of the seasonal moving average model, ${(1-{B}^{S})}^{D}$ is the component of the seasonal differences, ${a}_{t}$ is white noise^16,17^. In this study, the SARIMA model was constructed with outpatient data from 2015 to 2018. Based on the monthly outpatient visits from 2015 to 2018, we used the IBM Statistical Package for the Social Sciences (SPSS) Expert Modeler to find the appropriate model parameters.

Expert Modeler can combine data to automatically select the optimal model^11^. The parameters of the SARIMA model (p, d, q, P, D, Q, and s) were determined. The white noise of the residual series was diagnosed by the Ljung-Box test. The parameters should be adjusted until the residual sequence of an appropriately fitted model is white noise. The stationary ${R}^{2}$, coefficient of determination ${R}^{2}$, mean absolute percentage error (MAPE), and normalized Bayesian information criterion (BIC) were employed to diagnose an optimal SARIMA model. The best model should have the highest stationary ${R}^{2}$, ${R}^{2}$, and the lowest MAPE, BIC^18^.

To improve the fit and predictive power of the model, meteorological environmental factors were added to the SARIMA model. Spearman’s rank correlations were used to analyze the relationship between meteorological environmental factors and outpatient visits. Statistically significant variables and correlation coefficients greater than 0.4 were selected as covariates of the SARIMA model separately.

The SARIMA model with covariates is also known as the SARIMAX model (or seasonal ARIMAX). The SARIMAX model is based on SARIMA, and X are exogenous factors. The equation for SARIMAX is:

2 $${Y}_{t}=\frac{{\theta }_{q}(B){\Theta }_{Q}({B}^{S}){a}_{t}}{{\upphi}_{P}({B}^{S}){\varphi }_{p}(B){(1-B)}^{d}{(1-{B}^{S})}^{D}}+X$$

where *X* represents the univariate or multivariate exogenous variables or called covariates. The other parameters are the same as described in Eq. (1) above^19,20^. In this study, SARIMAX models were developed based on spearman’s results, and the optimal model was selected by comparing the values of stationary ${R}^{2}$, ${R}^{2}$, MAPE, and BIC for different models. Data from 2019 as validation of the optimal model. The predicted values of the model were compared with the actual values for 2019 to verify the predictive power of the model.

For the model to be practical, we used the meteorological environmental data for the average of the corresponding months from 2016 to 2018 to predict the monthly outpatient visits for 2019. Compared with actual values for 2019 to verify whether the mean of past years could be used as a covariate to predict outpatient visits.

To calculate the loss to the outpatient visits caused by the COVID-19 outbreak, we used this SARIMAX model with covariates to estimate the number of hospital outpatient visits lost between January 2020 and July 2021.

The time series data analysis was performed using SPSS 25.0 and we used the packages of “tseries” and “ggplot2” of R 4.1.0 (the R Core Team, Vienna, Austria) (https://cran.r-project.org/) to graph drawing. P < 0.05 was used as a criterion of significance.

### Ethics approval and informed consent

This study has been approved by the Ethics Committee of the First People’s Hospital of Kunshan (no. 2020-03-046-K01), and it complied with the Declaration of Helsinki. Patient information was initially recorded for hospital quality improvement. The informed consent requirement was waived by the Ethics Committee of the First People’s Hospital of Kunshan due to the retrospective nature of the study.

## Results

### Statistical description

**Figure 1:**
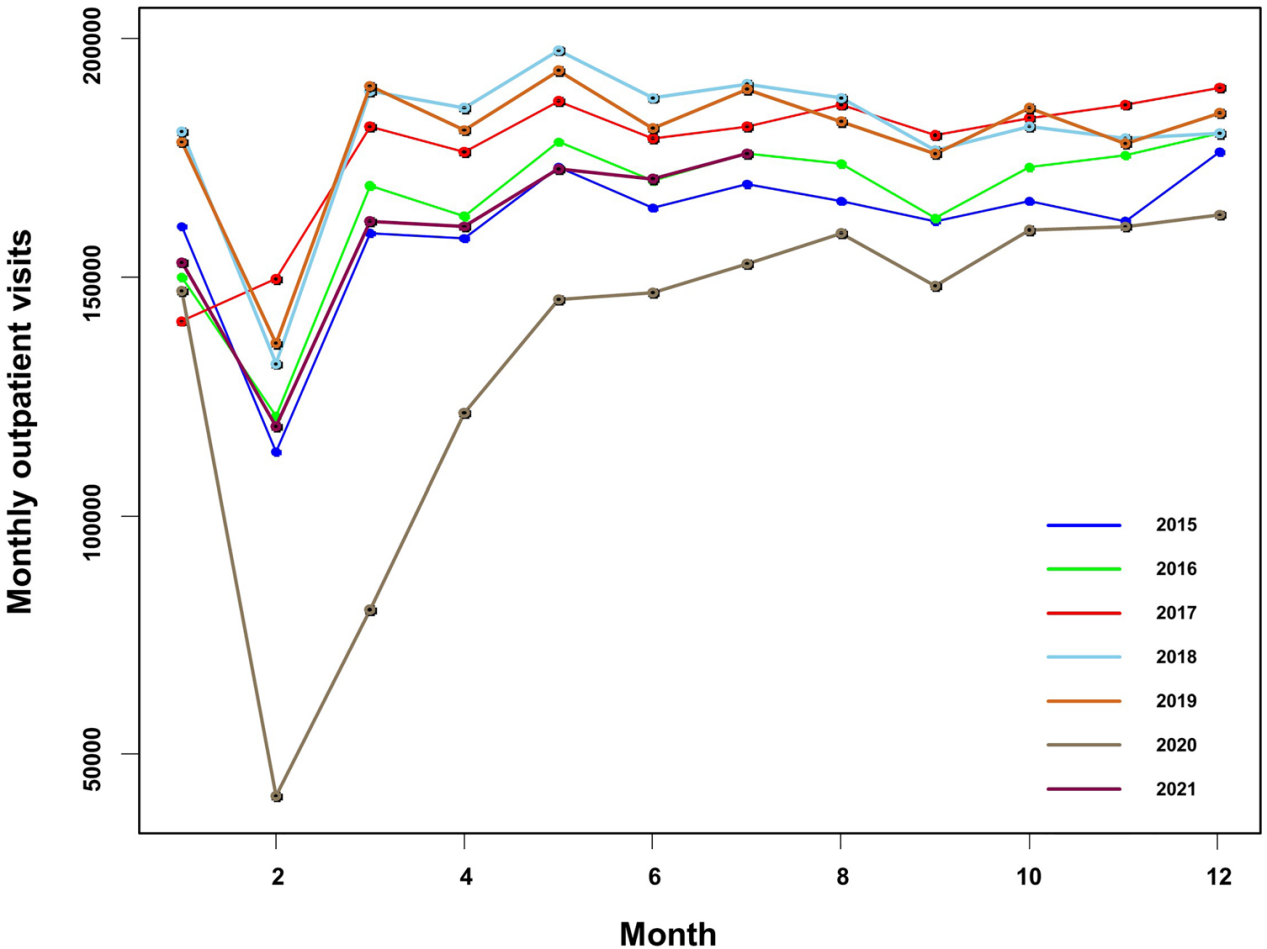
Monthly outpatient visits from January 2015 to July 2021.

**Figure 2:**
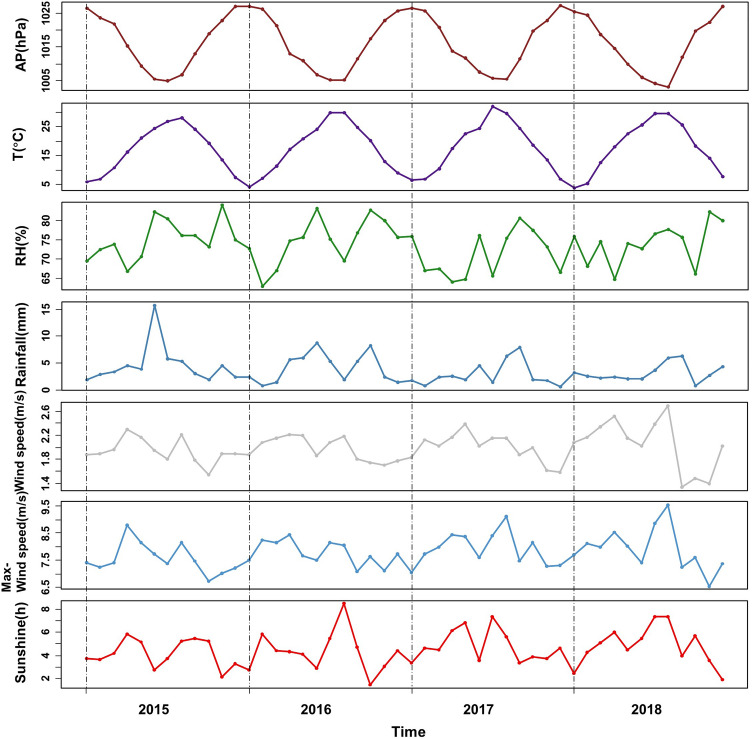
Monthly average meteorological factors from 2015 to 2018.

**Figure 3:**
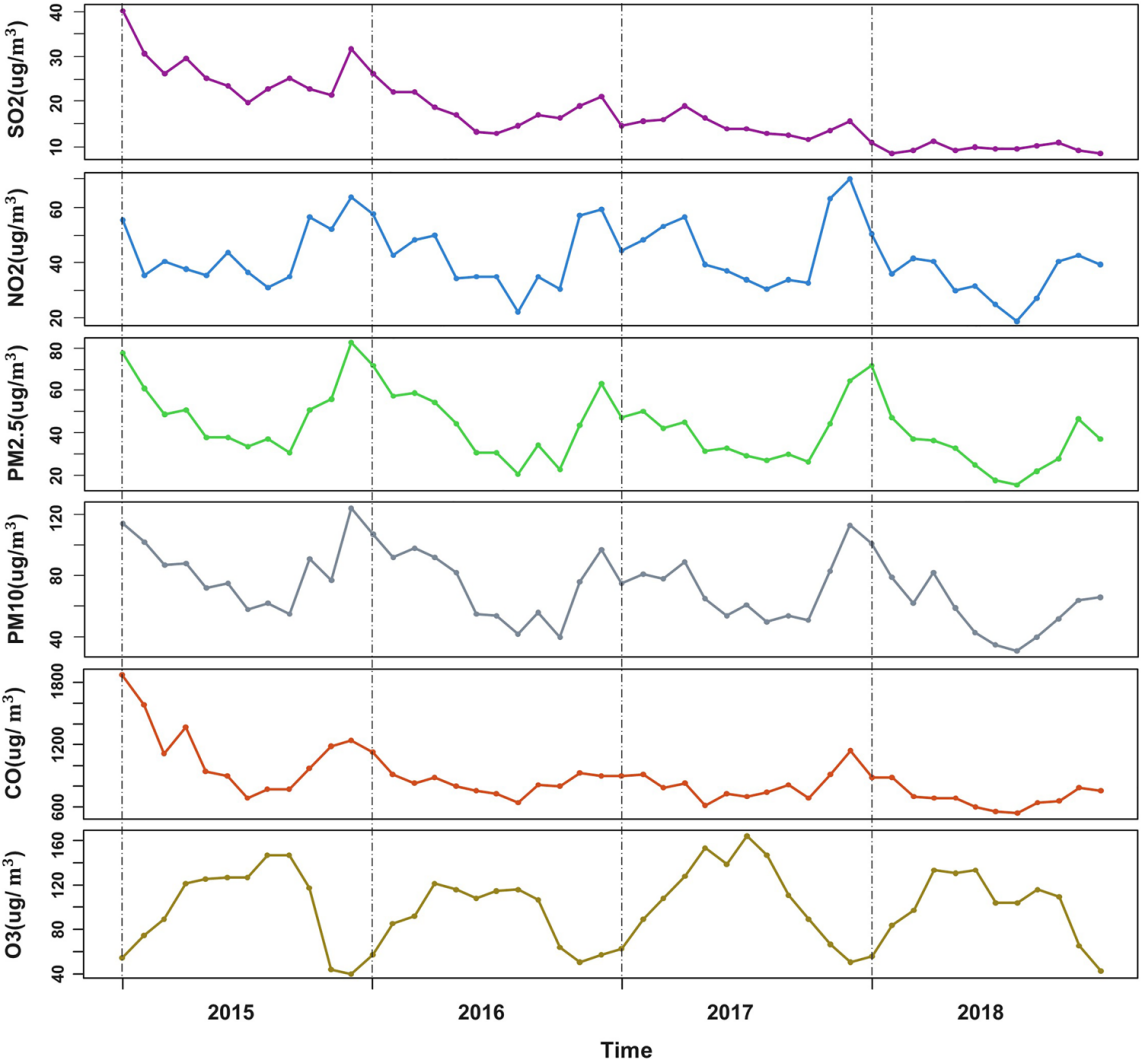
Monthly average air pollutant concentrations from 2015 to 2018.

The monthly outpatient visits from January 2015 to July 2021 were collected. A total of 13, 108, 742 cases in the last 79 months. The monthly trend of outpatient visits was shown in Fig. 1. As can be seen, Monthly outpatient visits have increased each year between 2015 and 2018. The monthly outpatient visits in 2019 were essentially the same as those in 2018. Monthly hospital outpatient visits tend to stabilize. The COVID-19 outbreak in early 2020 had a profound impact on hospital outpatient visits. As of July 2021, monthly outpatient visits have not returned to pre-COVID-19 outbreak levels. Every year, the number of outpatient visits in February decreases to a certain extent, considering that February is the Chinese Lunar New Year and Chinese people will be less likely to visit the doctor. Because outpatient visits from January 2020 onwards were heavily influenced by the COVID-19 epidemic, we only used data from 2015 to 2019 to build our prediction models. Data from 2015 to 2018 were used to find model covariates and built the model, with actual data from 2019 for validation. There was a total of 10, 368, 828 monthly outpatient visits from 2015 to 2019. Figure 2. shows the monthly averages of meteorological factors (atmosphere pressure, temperature, relative humidity, rainfall, 2-min average wind speed, maximum wind speed, and sunshine length) from 2015 to 2018. Temperature, humidity, and sunshine length were highest in the summer months (June–September). Figure 3. shown the monthly average $\text{SO}_{2}$, $\text{NO}_{2}$, ${PM}_{2.5}$, ${PM}_{10}$, $\text{CO}$, and $\text{O}_{3}$ concentration from 2015 to 2018. $\text{SO}_{2}$, $\text{NO}_{2}$, ${PM}_{2.5}$, and ${PM}_{10}$ showed the highest concentrations during the winter. $\text{CO}$ concentrations were decreased annually since 2015. $\text{O}_{3}$ concentrations were highest in summer months.

The summary statistics for monthly meteorological factors, monthly outpatient visits, and the results of Spearman’s rank correlation analysis from 2015 to 2018 were described in Table 1. The averages of atmosphere pressure, temperature, relative humidity, rainfall, 2-min average wind speed, maximum wind speed, and sunshine length were 1016.20 ± 8.19 hPa, 17.62 ± 8.49 °C, 73.76 ± 5.59%, 3.68 ± 2.71 mm, 1.99 ± 0.28 m/s, 7.79 ± 0.62 m/s, 4.56 ± 1.50 h. The summary statistics for monthly environmental factors and the results of Spearman’s rank correlation analysis from 2015 to 2018 were described in Table 2. The monthly average of $\text{SO}_{2}$, $\text{NO}_{2}$, ${PM}_{2.5}$, ${PM}_{10}$, $\text{CO}$, and $\text{O}_{3}$ concentration were 17.40 ± 7.13 ${\upmu {\text{g}}/\text{m}}^{3}$, 41.58 ± 11.54 ${\upmu {\text{g}}/\text{m}}^{3}$, 42.19 ± 15.93 ${\upmu {\text{g}}/\text{m}}^{3}$, 71.80 ± 22.50 ${\upmu {\text{g}}/\text{m}}^{3}$, 872.04 ± 254.76 ${\upmu {\text{g}}/\text{m}}^{3}$, 99.49 ± 33.51 ${\upmu {\text{g}}/\text{m}}^{3}$. Atmosphere pressure, temperature, $\text{SO}_{2}$, ${PM}_{2.5}$, ${PM}_{10}$, $\text{CO}$ concentration had statistical significance with the outpatient visits. The correlation coefficient of $\text{SO}_{2}$, ${PM}_{2.5}$, and $\text{CO}$ concentration was greater than 0.4.

**Table 1:** Summary statistics for monthly meteorological factors, monthly outpatient visits, and the results of Spearman’s rank correlation analysis from 2015 to 2018.

	Mean ± SD	Min	Max	Quartile			Correlation coefficient	
				P (25)	P (50)	P (75)	${r}_{s}$	P
Monthly outpatient visits	171,094 ± 17,529	113,283	197,685	162,374	176,080	181,732	–	–
Monthly average atmosphere pressure (hPa)	1016.20 ± 8.19	1003.08	1027.49	1007.86	1016.43	1023.69	− 0.295	P < 0.05
Monthly average temperature (℃)	17.62 ± 8.49	3.98	32.20	9.37	18.36	24.52	0.338	P < 0.05
Monthly average relative humidity (%)	73.76 ± 5.59	62.97	83.93	68.59	74.85	76.75	0.007	P > 0.05
Monthly average rainfall (mm)	3.68 ± 2.71	0.59	15.71	1.95	2.66	5.27	− 0.100	P > 0.05
Monthly 2-min average wind speed (m/s)	1.99 ± 0.28	1.34	2.70	1.81	2.03	2.17	0.161	P > 0.05
Monthly average maximum wind speed (m/s)	7.79 ± 0.62	6.55	9.56	7.37	7.69	8.16	0.242	P > 0.05
Monthly average sunshine length (h)	4.56 ± 1.50	1.48	8.46	3.64	4.44	5.50	0.267	P > 0.05

**Table 2:** Summary statistics for monthly environmental factors and the results of Spearman’s rank correlation analysis from 2015 to 2018.

	Mean ± SD	Min	Max	Quartile			correlation coefficient	
				P (25)	P (50)	P (75)	${r}_{s}$	P
Monthly average $\text{SO}_{2}$ concentration (${\upmu {\text{g}}/\text{m}}^{3}$)	17.40 ± 7.13	8.64	40.23	11.45	15.94	22.28	− 0.628	P < 0.01
Monthly average $\text{NO}_{2}$ concentration (${\upmu {\text{g}}/\text{m}}^{3}$)	41.58 ± 11.54	19.10	70.42	33.88	39.39	50.04	− 0.209	P > 0.05
Monthly average ${PM}_{2.5}$ concentration (${\upmu {\text{g}}/\text{m}}^{3}$)	42.19 ± 15.93	15.29	82.35	30.45	38.03	50.88	− 0.478	P < 0.01
Monthly average ${PM}_{10}$ concentration (${\upmu {\text{g}}/\text{m}}^{3}$)	71.80 ± 22.50	30.42	123.65	53.88	72.82	88.65	− 0.386	P < 0.01
Monthly average $\text{CO}$ concentration (${\upmu {\text{g}}/\text{m}}^{3}$)	872.04 ± 254.76	548.40	1874.20	697.60	806.65	923.43	− 0.626	P < 0.01
Monthly average $\text{O}_{3}$ concentration (${\upmu {\text{g}}/\text{m}}^{3}$)	99.49 ± 33.51	40.16	164.35	64.77	107.05	125.64	0.181	P > 0.05

### Model construction

We used Expert Modeler in SPSS to find the appropriate model parameters. The monthly incidence showed a seasonal trend with a seasonal cycle of 12 months. So, SARIMA ${(\mathrm{p},\mathrm{ d},\mathrm{ q}) (\mathrm{P},\mathrm{ D},\mathrm{ Q})}_{s}$ model was used. The Expert Modeler in SPSS determined the parameters of the model. $\mathrm{SARIMA}{(0, 1, 1) (0, 1, 0)}_{12}$ model is selected with MAPE (4.446), normalized BIC (18.73), stationary ${R}^{2}$(0.46) and ${R}^{2}$(0.55). The series of residuals are white noise based on the Ljung-Box test (P = 0.943), which meets the model evaluation criteria.

### SARIMAX model with meteorological environmental factors

Atmosphere pressure, temperature, $\text{SO}_{2}$, ${PM}_{2.5}$, ${PM}_{10}$, $\text{CO}$ concentration had statistical significance with the outpatient visits. The correlation coefficient of $\text{SO}_{2}$, ${PM}_{2.5}$, and $\text{CO}$ concentration was greater than 0.4. Therefore, they were added as covariates into SARIMA ${(0, 1, 1) (0, 1, 0)}_{12}$ model to assess whether to improve the fit and predictive power, respectively. A valuable predictor can increase the stationary ${R}^{2}$ and ${R}^{2}$ of the model. When atmosphere pressure, temperature, $\text{SO}_{2}$, ${PM}_{2.5}$, ${PM}_{10}$, $\text{CO}$ concentration as covariates, the stationary ${R}^{2}$ of the model was 0.56 and the ${R}^{2}$ was 0.63. The MAPE of the model was 4.305, normalized BIC was 19.47. When $\text{SO}_{2}$, ${PM}_{2.5}$, and $\text{CO}$ as covariates, the stationary ${R}^{2}$ of the model was 0.60 and the ${R}^{2}$ was 0.67. The MAPE of the model was 3.793 and the normalized BIC was 18.97. As the results, the model with the covariates of $\text{SO}_{2}$, ${PM}_{2.5}$, and $\text{CO}$ was best fitted to the time series. The result of the Ljung-Box test also indicated that the residual error of the optimal model was white noise.

**Figure 4:**
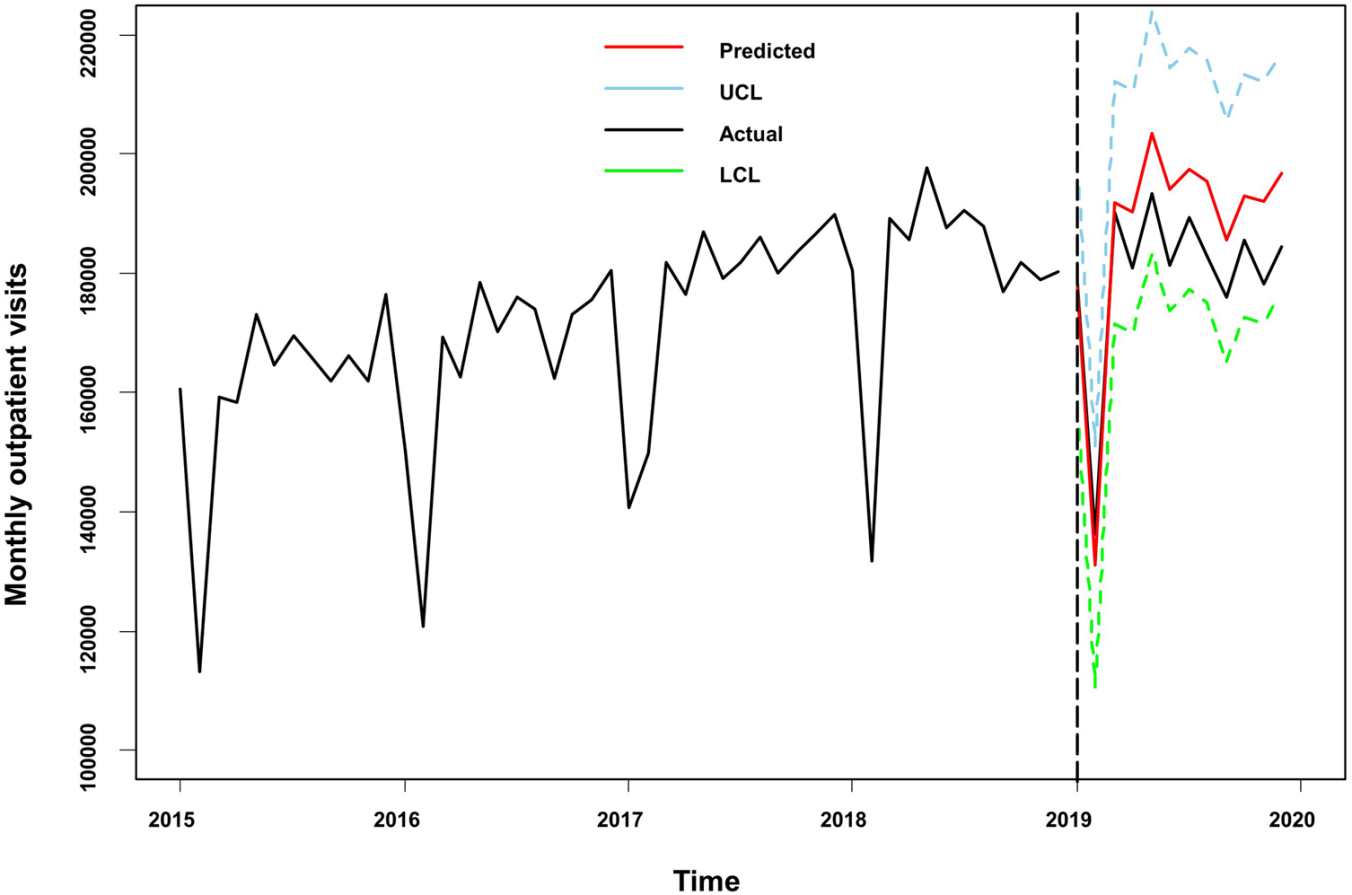
Prediction fitting of SRIMAX model for outpatient visits from January to December 2019. LCL, lower confidence interval; UCL, upper confidence interval.

According to the above analysis results, the SARIMA ${(0, 1, 1) (0, 1, 0)}_{12}$ model with the covariates of $\text{SO}_{2}$, ${PM}_{2.5}$, and $\text{CO}$ was used to predict the monthly outpatient visits in 2019. The model was also validated using data from 2019. Table 3 shows the predicted monthly outpatient visits for 2019. The relative error between the predicted and actual values for 2019 was 4.80%. The fitting prediction chart was shown in Fig. 4. The actual values were all within the 95% confidence interval of the prediction. The predicted trend was essentially the same as the actual values, the model fitted better in the first few months, and the predicted trend was essentially the same as the actual values.

**Table 3:** Prediction results of the SARIMAX model for outpatient visits from January to December 2019.

The year 2019	Jan	Feb	Mar	Apr	May	Jun	Jul	Aug	Sept	Oct	Nov	Dec
Actual value	178,355	136,162	190,283	180,753	193,458	181,247	189,379	182,779	175,834	185,584	178,152	184,325
Predicted value	177,584	130,998	191,807	190,218	203,505	194,056	197,458	195,378	185,482	192,889	191,913	196,806
Lower 95% CI	157,358	110,769	171,575	169,984	183,269	173,818	177,219	175,137	165,240	172,646	171,669	176,561
Upper 95% CI	197,811	151,228	212,039	210,452	223,741	214,294	217,698	215,618	205,725	213,133	212,158	217,052
The absolute value of error	771	5164	1524	9465	10,047	12,809	8079	12,599	9648	7305	13,761	12,481
Relative error	0.43%	3.79%	0.80%	5.24%	5.19%	7.07%	4.27%	6.89%	5.49%	3.94%	7.72%	6.77%
Average error	4.80%

**Figure 5:**
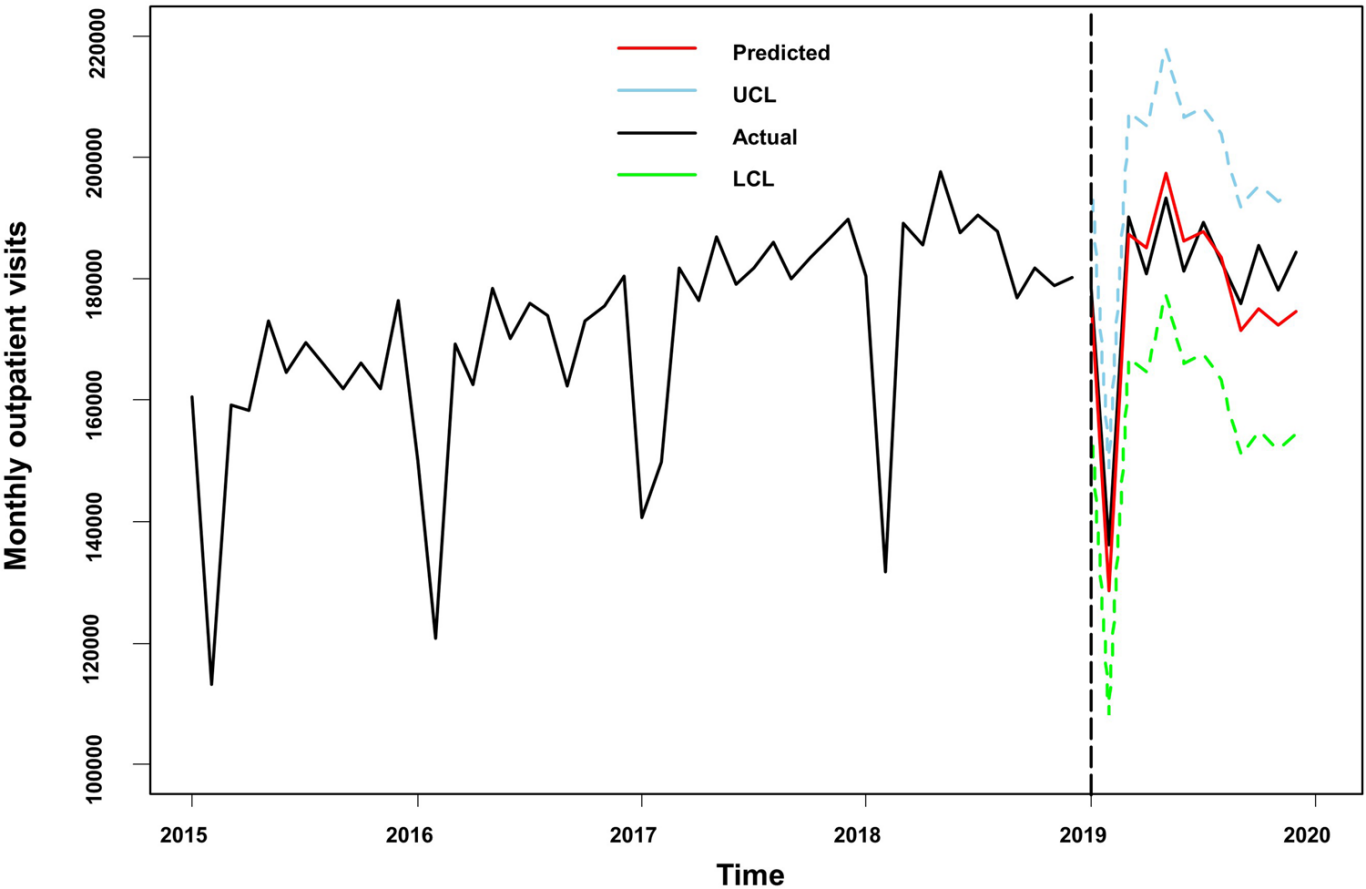
Prediction fitting of SRIMAX model for outpatient visits from January to December 2019 (concentration for the corresponding months from 2016 to 2018 as covariates). LCL, lower confidence interval; UCL, upper confidence interval.

For the model to have the ability to predict future outpatient visits. For the covariates, we used the average of the corresponding months from 2016 to 2018 to predict the monthly outpatient visits in 2019. Table 4 shows the predicted values. The relative error between the predicted and actual values for 2019 was 2.77%. The fitting prediction chart was shown in Fig. 5, the model fitted well with the time series of monthly outpatient visits. Covariates using the monthly averages over the past three years also provided a good predictor of outpatient visits.

**Table 4:** Prediction results of the SARIMAX model for outpatient visits from January to December 2019 (covariates data using the average of the corresponding months from 2016 to 2018).

The year 2019	Jan	Feb	Mar	Apr	May	Jun	Jul	Aug	Sept	Oct	Nov	Dec
Actual value	178,355	136,162	190,283	180,753	193,458	181,247	189,379	182,779	175,834	185,584	178,152	184,325
Predicted value	176,316	128,706	187,285	185,026	197,430	186,269	187,813	183,599	171,453	175,099	172,376	174,678
Lower 95% CI	156,090	108,477	167,053	164,792	177,193	166,031	167,574	163,358	151,211	154,856	152,132	154,432
Upper 95% CI	196,543	148,936	207,517	205,260	217,666	206,507	208,053	203,840	191,695	195,343	192,620	194,923
The absolute value of error	2039	7456	2998	4273	3972	5022	1566	820	4381	10,485	5776	9647
Relative error	1.14%	5.48%	1.58%	2.36%	2.05%	2.77%	0.83%	0.45%	2.49%	5.65%	3.24%	5.23%
Average error	2.77%

We used $\mathrm{SARIMA}{(0, 1, 1) (0, 1, 0)}_{12}$ model with covariates to estimate the number of hospital outpatient visits lost between January 2020 and July 2021. Tables 5 and 6 showed the prediction values for 2020 and January to July 2021, respectively. The COVID-19 outbreak caused about 613, 299 outpatient visits in the year 2020. And from January to July 2021, the COVID-19 outbreak resulted in a loss of approximately 181, 338 outpatient visits.

**Table 5:** The model predicted values and 95% confidence intervals for 2020.

The year 2020	Jan	Feb	Mar	Apr	May	Jun	Jul	Aug	Sept	Oct	Nov	Dec
Actual value	147,112	41,368	80,346	121,662	145,272	146,677	152,858	159,268	148,064	159,970	160,501	163,174
Predicted value	182,819	144,621	198,445	189,698	203,260	191,205	198,920	189,758	181,600	189,323	181,331	188,591
Lower 95% CI	165,266	127,068	180,891	172,144	185,705	173,650	181,366	172,204	164,045	171,768	163,776	171,037
Upper 95% CI	200,372	162,175	215,999	207,252	220,814	208,760	216,475	207,313	199,154	206,877	198,885	206,145

**Table 6:** The model predicted values and 95% confidence intervals from January to July 2021.

The year 2021	Jan	Feb	Mar	Apr	May	Jun	Jul
Actual value	153,141	118,648	161,868	160,601	172,810	170,616	175,958
Predicted value	187,038	146,684	197,943	186,984	198,352	185,864	192,115
Lower 95% CI	161,991	121,637	172,896	161,938	173,306	160,818	167,070
Upper 95% CI	212,085	171,731	222,989	212,030	223,398	210,909	217,160

## Discussion

The pressure on outpatient clinics is increasing every year due to the increase in the number of patients and the growing complexity of health conditions. Effective forecasting of outpatient visits is beneficial to anticipate and prevent medical resource shortages. Accurate forecasting of hospital outpatient visits is beneficial for the reasonable planning and allocation of healthcare resources to meet medical demands.

In this study, we explored the relationship between seven meteorological factors, six environmental pollutants, and outpatient visits. We not only analyzed the impact of meteorological environmental factors on outpatient visits but also developed a model that could predict monthly outpatient visits. Our results shown $\text{SO}_{2}$, ${PM}_{2.5}$, and $\text{CO}$ concentration had a more important relationship with monthly outpatient visits. The SARIMAX model $\mathrm{SARIMA}{(0, 1, 1) (0, 1, 0)}_{12}$ incorporated with these factors performed well in the prediction of outpatient visit. The validation in comparison with the actual values showed that our model had a better prediction with the relative error between the predicted and actual values for 2019 being 4.80% and the actual values were all within the 95% confidence interval of the prediction. We used the average of data from the previous three year’s meteorological environmental factors as covariates in our predictions to make our model more practically tractable. Our findings would be beneficial to the rational allocation of medical resources.

The ARIMA model, which is a time domain method, is considered one of the most useful models for seasonal time series prediction^21,22^. This is a very practical method because it allows the analysis not only of the outcome variables but also of the factors that affect them. Therefore, it is often applied in the prediction and analysis of influencing factors^10^. The time series model plays an important role in forecasting and is used to explore the effects of meteorological, environmental and socio-economic factors on outcomes while providing forecasts to inform management policy development.

According to the results of Spearman’s rank correlation analysis, atmosphere pressure, temperature, $\text{SO}_{2}$, ${PM}_{2.5}$, ${PM}_{10}$, $\text{CO}$ concentration were found to be correlated with the monthly outpatient visits. Among them, $\text{SO}_{2}$, ${PM}_{2.5}$, and $\text{CO}$ concentration, have a stronger relationship with monthly outpatient visits. More recently, research evidence has indicated that $\text{SO}_{2}$, ${PM}_{2.5}$, and $\text{CO}$ concentration had related to hospital outpatient visits. $\text{SO}_{2}$ concentration is correlated with outpatient visits of asthma^23^. A significant association between ambient ${PM}_{2.5}$ levels and outpatient visits in child with respiratory diseases^24^. $\text{CO}$ increased the total outpatient visits and $\text{CO}$ exerted adverse effect on respiratory, cardiovascular, genitourinary, gastrointestinal and neuropsychiatric diseases^25^.

During the COVID-19 epidemic, the government took preventive measures including the prohibition of leaving home unless necessary, to reduce hospital outpatient visits to prevent cross-infection, resulting in a rapid decrease in hospital outpatient visits^26–29^. Not only did the number of outpatient visits drop sharply in the pre-epidemic period, but the number of outpatient visits is now somewhat lower than before the outbreak. In our study, we projected a loss of approximately 794, 637 outpatient visits to the hospital between January 2020 and July 2021. To satisfy the needs of patients who cannot be seen in person due to the COVID-19 outbreak, hospitals may consider telemedicine services. Telemedicine tools include simple phone calls, the use of e-mails or text messages, and video visits. Telemedicine is associated with comparable outcomes and offers greater efficiency and service for patients^30^. A retrospective cohort study in the United States showed telemedicine services did not offset the reduction in outpatient visits; however, it did compensate for the reduction in outpatient visits^31^. In terms of whether reduced access to in-person care and increased telemedicine services affected patients' conditions, a study on the management of type 2 diabetes (T2D) in older U.S. veterans showed no observed effect of telemedicine visits on T2D control or short-term T2D-related outcomes^32^. In a study of the effectiveness of telemedicine visits in reducing 30-day readmissions in patients with heart failure during the COVID-19 pandemic, it was shown that patients with heart failure who received outpatient follow-up either via telemedicine or in-person had better outcomes than those who received no follow-up^33^. A telehealth model for outpatients with heart failure allowed for distanced encounters without increases in subsequent acute care or mortality^34^. Telemedicine services also have challenges in physical exams, particularly otoscopy, nasal endoscopy, and nasolaryngoscopy. Sufficient information is needed from patients and families to overcome these difficulties^35^. Telemedicine has many advantages and benefits, and can also be used to relieve medical stress after COVID-19^36^.

We acknowledge that our research has some limitations. First, this study only used monthly outpatient visits to Kunshan City to evaluate the performance of the built models and to find important variables as meaningful signs for outpatient visits. The findings of this study could not be directly extended to other countries’ outpatient visits. Second, in addition to meteorological environmental factors, the factors that affect outpatient visits such as seasons, and holidays were not taken into consideration, these factors may have an impact on the relationship between meteorological environmental factors and outpatient visits. Third, it is a lack of discussions and analyses on alternative forecasting models and reasons why they are not applicable. Another limitation of this study was that outpatient visit was not divided into specific outpatient departments such as surgery, dermatology, etc. Nevertheless, our study confirms that $\text{SO}_{2}$, ${PM}_{2.5}$, and $\text{CO}$ concentration have important effects on hospital outpatient visits. The model built has great predictability. Can provide a reference for hospital management.

In conclusion, our study suggests that atmosphere pressure, temperature, $\text{SO}_{2}$, ${PM}_{2.5}$, ${PM}_{10}$, $\text{CO}$ concentration have a significant impact on outpatient visits. Of these, $\text{SO}_{2}$, ${PM}_{2.5}$, and $\text{CO}$ concentration have a more important relationship with outpatient visits. The model we built also allows for the prediction of monthly outpatient visits by using meteorological environmental data from the previous three years. The model is relatively simple and has low computational intensity. Also, the results can be used to support the decisions of outpatient resource planning and scheduling. Help hospital managers to make the right decisions to meet the expected healthcare demand effectively and timely.

## Data Availability

The datasets used and/or analyzed in this study are available from the corresponding author upon reasonable request.

## Abbreviations

ARIMA: Autoregressive integrated moving average model
DLNM: Distributed lag nonlinear model
GLM: Generalized linear model
SARIMA: Seasonal autoregressive integrated moving average model
SARIMAX: Seasonal autoregressive integrated moving average model incorporating covariates
AR: Autoregressive
MA: Moving average
BIC: Bayesian information criterion
MAPE: Mean absolute percent error
UCL: Upper confidence limit
LCL: Lower confidence limit
COVID-19: Corona Virus Disease 2019
